# Intermedin prevents acute heart failure following acute kidney injury by alleviating inflammatory responses

**DOI:** 10.1080/0886022X.2025.2610795

**Published:** 2026-01-12

**Authors:** Yuyin He, Shuhui Jing, Keyu Zhang, Kaixin Yao, Jie Niu, Yuan Ge, Xiaole Su, Xi Qiao

**Affiliations:** ^a^ Department of Nephrology, Second Hospital of Shanxi Medical University, Taiyuan, People’s Republic of China; ^b^ Shanxi Kidney Disease Institute, Taiyuan, People’s Republic of China; ^c^ Kidney Research Center of Shanxi Medical University, Taiyuan, People’s Republic of China

**Keywords:** Acute kidney injury, acute heart failure, inflammatory response, intermedin, ischemia-reperfusion injury

## Abstract

Acute heart failure (AHF) is a common complication of acute kidney injury (AKI), which leads to a poor prognosis in AKI patients. The pathogenesis of AHF is closely related to cardiac inflammatory responses. Intermedin (IMD) has been demonstrated to attenuate cardiac inflammation, however, its role in AHF following AKI (AKI-AHF) remains to be elucidated. We aimed to investigate the effects of IMD on AKI-AHF and to elucidate the underlying mechanisms. We demonstrated that AKI induced by bilateral kidney ischemia-reperfusion injury (IRI) upregulated IMD and its receptors in the kidneys and heart. Following kidney IRI, AHF was more severe in IMD knockout (IMD^−/−^) mice compared with wild-type littermates, indicating IMD attenuates AKI-AHF. To clarify whether IMD has a direct effect on the heart, we used bilateral nephrectomy (BNX)-induced AKI model to exclude the influence of the degree of AKI on AHF. The results also showed that the cardiac injury in IMD^−/−^ mice was more severe than that in IMD^+/+^ mice, indicating that IMD has a direct protective effect on the heart. Serum inflammatory mediators were elevated more significantly in IMD^−/−^ mice compared with IMD^+/+^ mice after AKI. Additionally, IMD^−/−^ mice exhibited enhanced activation of NF-κB, as well as increased expression of inflammatory mediators in cardiac tissue following AKI. In summary, IMD protects AKI-AHF through the reduction of both localized cardiac inflammation and systemic inflammatory responses following kidney IRI. Upregulation of cardiac IMD expression may represent a viable approach to attenuate AKI-AHF and improve the prognosis of AKI, warranting further investigation.

## Graphical Abstract

**Figure:**
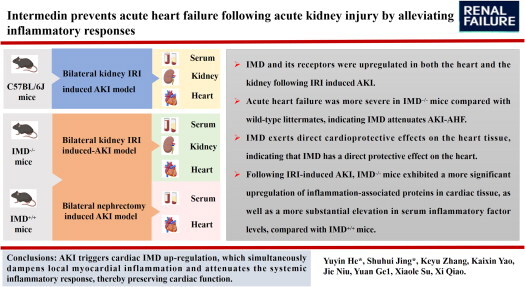

## Introduction

Acute kidney injury (AKI) is a clinical syndrome characterized by rapid deterioration of kidney function within a short period, resulting from various etiological factors [1,2]. Epidemiological studies indicate that 10-15% of hospitalized patients develop AKI, with incidence rates exceeding 50% in intensive care units [3]. As a systemic disorder, AKI frequently induces secondary damage to distant organs including the heart, lungs, and liver, which constitutes a major determinant to its persistently high mortality rates [4,5]. Current research demonstrates that heart failure, particularly acute heart failure (AHF) categorized as type 3 cardiorenal syndrome (CRS), represents the most prevalent cause of mortality within one year post-hospitalization for AKI patients, while also exerting detrimental effects on clinical prognosis [6,7]. Consequently, preventing AHF following AKI holds critical significance in both lowering mortality risks and enhancing clinical outcomes in patients with AKI.

The massive release of inflammatory mediators is the primary cause of the distant organ effects induced by AKI [8]. The development of AKI initiates widespread inflammatory activation, which extends from the circulatory system to remote organ systems, thereby leading to subsequent tissue injury and organ failure [9–11]. Kelly et al. demonstrated that localized myocardial inflammation following AKI acts as a critical pathogenic driver in the development of heart failure [12]. Pro-inflammatory cytokines including tumor necrosis factor-α (TNF-α), interleukin-1 (IL-1), and interleukin-6 (IL-6) exhibit close associations with the pathogenesis of heart failure [13]. Mechanistically, TNF-α and IL-1 activate the nuclear factor-κB (NF-κB) signaling pathway, upregulating vascular cell adhesion molecule-1 (VCAM-1) expression to facilitate leukocyte migration to inflammatory sites [14,15]. After binding to IL-6 receptor (IL-6R), IL-6 activates downstream signaling pathways, indirectly promoting the activation of NF-κB [16,17]. The activated NF-κB subsequently translocates to the nucleus, binding to specific κB sites to enhance transcription and expression of cytokines, adhesion molecules, and chemokines [18,19]. This positive feedback mechanism amplifies inflammatory responses, exacerbating cardiac injury and functional impairment. Therefore, suppressing the post-AKI inflammatory response can mitigate subsequent AHF, representing a crucial intervention target for improving AKI prognosis [12].

Intermedin (IMD) or adrenomedullin 2 (ADM2), an endocrine/paracrine small peptide, is primarily distributed in human tissues including the kidneys, heart, lungs, and brain [20,21]. IMD is expressed in multiple cell types, such as kidney tubular epithelial cells, glomerular mesangial cells, cardiomyocytes, cardiac fibroblasts, and cardiac vascular endothelial cells [22,23]. IMD exerts a wide range of biological effects through nonselective interactions with the calcitonin receptor (CRLR) and three receptor activity-modifying proteins (RAMP1/2/3) [24]. Previous studies have primarily investigated the protective roles of IMD in cardiovascular contexts such as myocardial ischemia-reperfusion injury, diabetic cardiomyopathy, and vascular calcification. These studies have highlighted IMD reduces inflammation and oxidative stress, suppresses apoptosis, and inhibits vascular smooth muscle cell remodeling *via* pathways including cAMP/PKA and PI3K/Akt cascades. However, the role of IMD in AKI-induced acute heart failure (AKI-AHF) remains poorly defined. AKI-AHF is a clinically significant yet mechanistically underexplored syndrome involving crosstalk between injured kidneys and the heart. To our knowledge, this study is the first to investigate whether IMD mitigates cardiac injury after AKI and to elucidate the underlying inflammation-related signaling pathways in this context, thereby offering new insights into the therapeutic potential of IMD in the cardiorenal syndrome. Based on this evidence, we hypothesize that IMD may prevent AKI-associated AHF by suppressing inflammatory reactions in cardiac tissue.

In this study, we first examined the expression of IMD and its receptors in kidney and cardiac tissues using the classical AKI model, the bilateral kidney ischemia-reperfusion injury (IRI) model. Subsequently, IMD gene knockout (IMD^−/−^) mice were employed to evaluate the functional role of IMD in AHF following AKI. To clarify whether IMD exerts direct cardioprotective effects, we utilized a bilateral nephrectomy (BNX)-induced AKI model to eliminate the influence of kidney injury severity on AHF development. Finally, we explored the regulatory effects IMD on inflammatory mediators in cardiac tissues and systemic circulation following AKI to elucidate its underlying protective mechanisms. This study is designed to investigate the impact and underlying mechanisms of IMD on AHF following AKI. It aims to identify novel therapeutic targets for the management of AHF in the context of AKI, and to propose innovative strategies to enhance clinical outcomes in patients with AKI.

## Materials and methods

### Experimental animals and grouping

Male C57BL/6J mice (8–10 weeks old, weighing 20–25 g), IMD^−/−^mice on C57BL/6 background, and wild-type (IMD^+/+^) mice were used in this study. All animal experiments were approved by the Animal Ethics Committee of the Second Hospital of Shanxi Medical University (Approval No. DW2024060). Animals were housed under controlled lighting and temperature conditions with free access to food and water. The three types of mice were randomly assigned to the sham-operated group, IRI group, or BNX group. Blood samples, cardiac tissues, and kidney tissues were collected from six mice per group at 24 h, 48 h, and 72 h after IRI, and at 24 h and 48 h after BNX.

### Establishment of animal models

Standardized Surgical Procedures: Following an 8 h fasting period mice were anesthetized with sodium pentobarbital (50 mg/kg, i.p.). Bilateral kidney pedicles were exposed through longitudinal incisions parallel to the vertebral column. Sham group: Animals underwent kidney artery isolation without vascular occlusion. IRI-AKI model: Kidney arteries were bilaterally clamped within 1 min using microvascular clips. Successful ischemia was confirmed by kidney color change from bright red to purple within 1–5 min. After 30 min ischemia, clips were removed and reperfusion was verified by restoration of kidney redness within 1–5 min. BNX-AKI model: Kidney pedicles were doubly ligated and transected for bilateral nephrectomy. Throughout procedures, core body temperature was maintained at 37 ± 0.5 °C using a heating pad. Postoperative care included layered wound closure, 0.5 mL warm saline (i.p.), and continuous monitoring until full ambulation recovery prior to transfer to SPF housing.

### Cardiac ultrasound examination

*In vivo* cardiac morphology was assessed in mice anesthetized with 2–2.5% isoflurane gas using transthoracic echocardiography with an ultrasound machine (Model Vevo-LAZR-X; FUJIFILM VisualSonics). The M-mode left ventricular internal diameter at end-systole (LVIDs) and end-diastole (LVIDd), left ventricular end-systolic volume (LVESV) and end-diastolic volume (LVEDV), anterior wall thickness at end-systole (LVAWs) and end-diastole (LVAWd), posterior wall thickness at end-systole (LVPWs) and end-diastole (LVPWd), stroke volume (SV), cardiac output (CO), left ventricular ejection fraction (LVEF), fractional shortening (LVFS), and left ventricular mass (LV Mass) were averaged from 3 to 5 beats.

### Specimen collection

After weighing and recording body weights, mice were anesthetized with 3% sodium pentobarbital (50 mg/kg, i.p.). Blood samples were collected *via* abdominal aortic puncture, followed by cardiac perfusion using 50 mL pre-cooled saline until hepatic blanching. Subsequently, heart and kidney tissues were harvested, rinsed with saline, and blotted dry. Organ morphology, size, and coloration were documented before tissue weighing. Calculated by comparing kidney weight to mouse body weight. The kidney/body weight ratio reflects changes in kidney size and weight and is an important indicator for assessing the pathological state of the kidneys. Blood samples were centrifuged at 4 °C (3,000 rpm, 15 min) to isolate serum, which was preserved at −20 °C. Kidney and cardiac tissue specimens were divided into aliquots and stored at −80 °C for subsequent analysis.

### Kidney function assessment

Blood samples were centrifuged at 3,000 rpm for 15 min to obtain serum. Serum creatinine (Scr) and blood urea nitrogen (BUN) levels in the blood samples were measured using creatinine (Nanjing Jiancheng, C011-2-1, Nanjing, China) and blood urea nitrogen assay kits (Nanjing Jiancheng, C013-2-1, Nanjing, China), respectively, following the manufacturer’s instructions. The absorbance of Scr and BUN was measured at 546 and 640 nm, respectively, using a colorimetric method. Final concentrations were calculated according to standard formulas.

### Enzyme-linked immunosorbent assay (ELISA)

The cardiac troponin-T (Tn-T), brain natriuretic peptide (BNP), TNF-α, and IL-6 levels in the serum of mice were identified by utilizing Mouse Tn-T ELISA kit (MM-44145M1, Jiangsu, China), BNP ELISA kit(MM-0060M1, Jiangsu, China), TNF-α ELISA kit(MM-0132M1, Jiangsu, China), and IL-6 ELISA kit(MM-0163M1, Jiangsu, China) according to the directions of the manufacturer.

### Histopathological examination

The kidney and heart tissue were fixed in a 4% formaldehyde solution and subjected to histological analysis *via* hematoxylin and eosin (H&E) staining and periodic acid-Schiff (PAS) staining. Kidney tissue injury was quantified in a fully blinded manner using the Paller’s scoring system. After whole-slide digitization, ten non-overlapping fields at 400× were randomly selected by a researcher unaware of group allocation. Each field was scored for tubular dilation, brush-border loss, cast formation, cellular flattening, and epithelial shedding; detailed criteria are provided in Supplementary Table 1. This protocol ensured unbiased evaluation of H&E staining sections. Cardiac damage was similarly assessed by examining cardiomyocyte morphology, myocardial fiber striations, and interstitial inflammatory infiltration.

### Immunohistochemical staining

Kidney tissue sections and cardiac tissue sections were baked at 67 °C for deparaffinization, followed by antigen retrieval using citrate buffer. The kidney sections were then incubated with anti-IMD primary antibody (Bioss, bs-2985R) at a dilution of 1:200 overnight at 4 °C, while cardiac tissue sections were incubated with anti-IMD primary antibody at a dilution of 1:400. Subsequently, sections were incubated with secondary antibody at room temperature for 1 h. Following DAB chromogenic development, all sections underwent hematoxylin counterstaining. Immunohistochemistry images were acquired with a whole-slide scanner and cropped at 400× magnification. Six random fields per section were quantified in GraphPad Prism version 10.1.2 by an investigator blinded to group allocation. Integrated staining intensity was recorded for each field and expressed as mean ± SD, ensuring objective and unbiased analysis.

### RT-qPCR

Three samples per group were randomly selected for quantitative real-time PCR (RT-qPCR). Target genes (IMD, CRLR, RAMP1, RAMP2, and RAMP3) were analyzed using primers listed in Table 1. The 25 μL reaction mixture contained: 12 μL TB Green Premix Ex Taq^™^ (Takara), 1 μL forward primer, 1 μL reverse primer, 2.5 μL cDNA template, and 8.5 μL nuclease-free water. Reaction conditions: Pre-denaturation at 95 °C for 30 s; cycling (40 cycles): 95 °C for 5 s, 60 °C for 30 s; melting curve analysis: 95 °C for 10 s, 65 °C for 5 s, then hold at 4 °C indefinitely. The 18S rRNA gene served as an internal reference, and relative mRNA expression levels were calculated using the 2^−ΔΔCt^ method based on Ct values.

**Table 1.** List of RT-qPCR primer sequences.

Oligo name	Sequence (5′to 3′)	
IMD	Forward	5′-TGATGAGACGACAGTTCCTACCC-3′
	Reverse	5′-CGCTCTGATTGCTGGCTTGTAG-3′
CRLR	Forward	5′-CATCCACGCCATTGCCAGAAG-3′
	Reverse	5′-TTTACCAACAAAGCAGCACAAATCG-3′
RAMP1	Forward	5′-GAGACTATTGGGAAGACGCTATG-3′
	Reverse	5′-GCTAGCTGATCCCAACTGAAA-3′
RAMP2	Forward	5′-GGGAAGATGGAAGACTACGAAACAC-3′
	Reverse	5′-GGTCGCTGTAATGCCTGCTAATC-3′
RAMP3	Forward	5′-AGAAGGTGGCTGTCTGGAAGTG-3′
	Reverse	5′-CCATCTCGGTGCAGTTAGTGAAG-3′
18SrRNA	Forward	5′-CGGACACGGACAGGATTGACAG-3′
	Reverse	5′-AATCGCTCCACCAACTAAGAACGG-3′

### Western blotting

Total protein was extracted from the kidneys and heart of mice using the RIPA lysis buffer. The SDS-polyacrylamide gel electrophoresis technique (SDS-PAGE) was employed to separate and purify the protein samples, which were subsequently transferred to a PVDF membrane. The membranes were incubated with primary antibodies overnight at 4 °C. HRP-bound secondary antibody bands were detected using ECL. Subsequently, protein densitometry analysis was performed with ImageJ software, with β-actin serving as an internal standard protein. The antibodies included those against IMD, CRLR, RAMP1, RAMP2, RAMP3, NF-κB p65, NF-κB p-p65, TNF-α, IL-6, IL-1β, and VCAM-1. The primary antibodies used are listed in Supplementary Table 2.

### Statistical analysis

Statistical analyses were performed using GraphPad Prism version 10.1.2. For datasets conforming to a normal distribution with homogeneity of variance, one-way or two-way analysis of variance (ANOVA) was applied. Welch’s ANOVA was utilized for datasets exhibiting non-normal distribution and/or heteroscedasticity. Tukey’s *post hoc* multiple comparison test was subsequently performed. Results were expressed as mean ± SD, based on at least three independent experiments and *p* < 0.05 was considered statistically significant.

## Results

### The expression levels of IMD and its receptor are significantly upregulated in both kidney and heart following AKI

**Figure 1.**
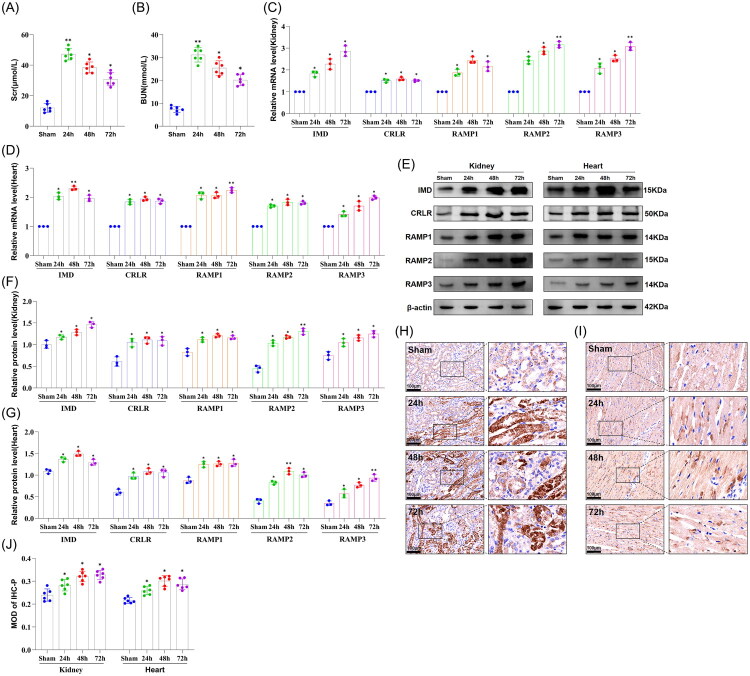
Expression levels of IMD and its receptors in the kidney and the heart of C57BL/6J mice following AKI. (A) Serum creatinine (Scr) levels; (B) Blood urea nitrogen (BUN) levels; (C) mRNA expression levels of IMD and its receptors in the kidney; (D) mRNA expression levels of IMD and its receptors in the heart; (E) Representative western blot images of IMD and its receptors; (F) Quantitative analysis of protein expression levels of IMD and its receptors in the kidney; (G) Quantitative analysis of protein expression levels of IMD and its receptors in the heart; (H) Immunohistochemical staining of IMD in the kidney; (I) Immunohistochemical staining of IMD in the heart; (J) Semi-quantitative analysis of IMD immunohistochemical staining. The data are presented as the mean ± SD. (*n* = 6/group). *p < 0.05 and **p < 0.01, IRI versus sham.

We investigated the expression of IMD and its receptors in the kidneys and heart of C57BL/6J mice following AKI using a bilateral kidney IRI model. Scr and BUN levels were significantly elevated in the 24 h, 48 h, and 72 h post-IRI groups compared to the sham group (*p* < 0.05; Figure 1A and B), confirming successful establishment of the AKI model. Notably, the 24 h group exhibited the most pronounced increases in Scr and BUN levels, indicating that kidney injury severity peaked at this time point.

Subsequently, RT-qPCR (Figure 1C and D) and Western blotting (Figure 1E–G) analyses revealed significant upregulation of IMD, CRLR, and RAMP1/2/3 mRNA and protein expression in both kidney and heart across all post-IRI time points compared to the sham group (*p* < 0.05). These findings suggest that IMD may play a role in injury and repair processes in the kidney and heart following kidney IRI. Immunohistochemical staining further validated the spatial distribution and intensity of IMD expression in the kidney and heart (Figure 1H–J). In the kidney, IMD is primarily localized to kidney tubular epithelial cells, with minor expression observed in glomerular mesangial cells. In the heart, IMD is mainly detected in cardiomyocytes and cardiac fibroblasts, while showing a minor presence in cardiac vascular endothelial cells. Collectively, these results demonstrate tissue-specific IMD distribution and its significant upregulation following IRI.

### IMD Alleviates AHF following AKI induced by kidney IRI

#### IMD Alleviates AKI induced by kidney IRI

**Figure 2.**
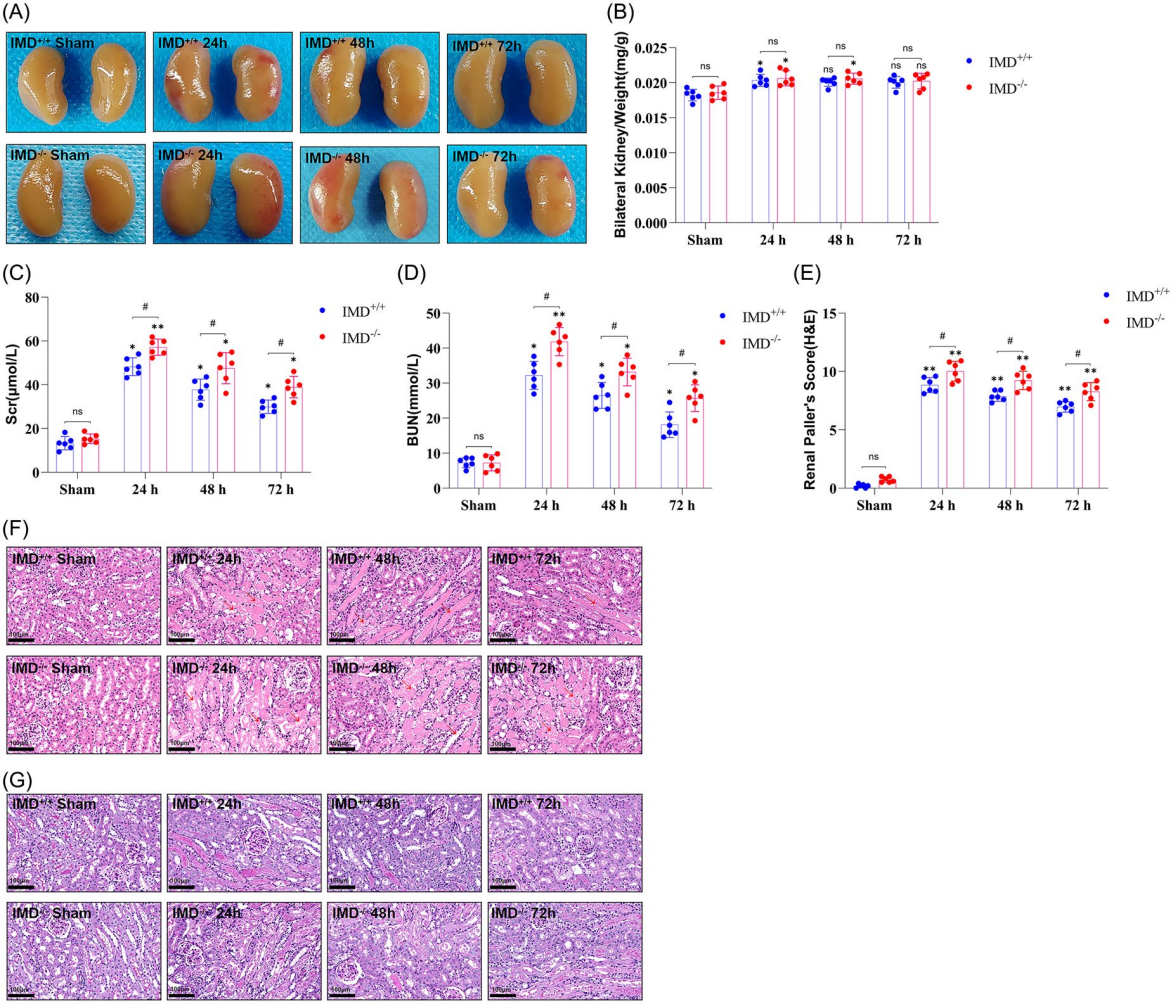
Renal injury in IMD^+/+^ and IMD^−/−^ mice after kidney ischemia-reperfusion injury. (A) Gross anatomical images of kidneys; (B) Kidney index (mg/g) calculated as bilateral kidney weight/body weight ratio; (C) Serum creatinine (Scr) levels; (D) Blood urea nitrogen (BUN) levels; (E) Paller’ s score based on H&E staining; (F) Representative H&E staining images showing kidney pathological changes (arrows indicate focal necrosis of renal tubular epithelial cells, brush border loss, tubular dilatation with protein casts and cellular debris in lumen, and inflammatory cell infiltration in interstitium); (G) PAS staining results of kidneys. The data are presented as the mean ± SD. (*n* = 6/group). ns: no significance; *p < 0.05 and **p < 0.01, IRI versus sham; ♯*p* < 0.05, IMD^−/−^ mice versus IMD^+/+^ mice at the same time point after IRI.

To investigate the role of up-regulated cardiac expression of IMD following AKI in the development of AHF after AKI, we induced kidney I/R injury in IMD^−/−^ mice. Initially, the genotypes of IMD^−/−^ and IMD^+/+^ mice were confirmed using established genotyping methods (Supplementary Fig. 1). Utilizing these mice, we investigated the biological role of IMD in AKI-AHF. Macroscopic examination of kidney tissues revealed no significant morphological differences between IMD^+/+^ sham and IMD^−/−^ sham groups (Figure 2A). In contrast, kidneys from mice subjected to kidney IRI at 24 h, 48 h, and 72 h exhibited increased size, uneven surface color, and capsular tension compared with sham-operated controls. No significant intergroup differences in kidney gross morphology were observed between IMD^−/−^ and IMD^+/+^ mice at corresponding time points. Similarly, the kidney-to-body weight ratio (mg/g) showed no significant difference between the IMD^−/−^ sham group and the IMD^+/+^ sham group. However, this ratio increased significantly at 24 h and 48 h post-IRI (*p* < 0.05; Figure 2B). Although the kidney-to-body weight ratio was numerically higher in IMD^−/−^ mice compared with IMD^+/+^ mice at the same time points, the difference lacked statistical significance (*p* > 0.05; Figure 2B). These results suggest that kidney IRI induces kidney congestion and edema.

Scr and BUN analyses demonstrated no significant differences between IMD^+/+^ and IMD^−/−^ sham groups (Figure 2C and D, Table 2, Supplementary Fig. 4, and Supplementary Table 4). However, both parameters were significantly elevated (*p* < 0.05) in IMD^+/+^ and IMD^−/−^ mice at all post-injury time points compared to sham controls, with peak values observed at 24 h. Notably, IMD^−/−^ mice exhibited more pronounced increases in Scr and BUN than their IMD^+/+^ counterparts at corresponding time points, suggesting that IMD mitigates kidney functional deterioration following IRI.

**Table 2.** Summary table of serum indicators in IRI-AKI and BNX-AKI mouse models.

Group	Indicators	IRI-AKI Model		BNX-AKI Model	
		IMD^+/+^ mice	IMD^−/−^ mice	IMD^+/+^ mice	IMD^−/−^ mice
Sham	Scr (μmol/L)	13.26 ± 3.02	15.31 ± 2.17	13.80 ± 2.97	14.31 ± 3.65
	BUN (mmol/L)	7.27 ± 1.42	7.23 ± 2.30	7.54 ± 2.25	8.02 ± 1.93
	Tn-T (ng/L)	182.95 ± 27.19	183.74 ± 19.09	181.66 ± 28.63	184.26 ± 21.78
	BNP (ng/L)	53.93 ± 13.02	55.91 ± 13.64	53.75 ± 14.17	54.38 ± 13.52
	TNF-α (ng/L)	210.13 ± 19.17	212.26 ± 25.21	–	–
	IL-6 (ng/L)	20.95 ± 3.88	22.00 ± 4.04	–	–
24 h	Scr (μmol/L)	48.24 ± 4.06^*^	57.11 ± 3.71^**♯^	59.88 ± 5.91^*^	60.14 ± 4.00^*^
	BUN (mmol/L)	32.23 ± 4.01^*^	41.83 ± 3.97^**♯^	44.06 ± 5.19^*^	44.21 ± 6.70^*^
	Tn-T (ng/L)	373.58 ± 27.09^*^	439.40 ± 32.33^*♯^	282.98 ± 23.04^*^	309.23 ± 18.80^*^
	BNP (ng/L)	117.81 ± 9.10^*^	145.28 ± 10.25^*♯^	102.90 ± 14.75^*^	123.49 ± 12.25^*^
	TNF-α (ng/L)	478.63 ± 15.36^*^	523.13 ± 23.00^**♯^	–	–
	IL-6 (ng/L)	136.43 ± 8.36^**^	151.05 ± 5.15^**^	–	–
48 h	Scr (μmol/L)	37.73 ± 4.80^*^	47.54 ± 7.11^*♯^	82.10 ± 8.18^**^	83.61 ± 7.27^**^
	BUN (mmol/L)	26.49 ± 3.68^*^	33.17 ± 3.93^*♯^	61.92 ± 4.76^**^	61.60 ± 5.34^**^
	Tn-T (ng/L)	439.87 ± 32.28^*^	505.74 ± 24.20^**♯^	377.83 ± 19.54^*^	423.29 ± 25.15^**♯^
	BNP (ng/L)	126.96 ± 11.64^*^	153.80 ± 9.27^**♯^	115.72 ± 9.55^*^	141.91 ± 9.12^**♯^
	TNF-α (ng/L)	456.07 ± 25.63^*^	508.15 ± 24.10^**♯^	–	–
	IL-6 (ng/L)	124.18 ± 5.95^**^	146.09 ± 7.01^**♯^	–	–
72 h	Scr (μmol/L)	29.93 ± 3.03^*^	38.88 ± 4.85^*♯^	–	–
	BUN (mmol/L)	18.13 ± 3.66^*^	25.69 ± 3.377^*♯^	–	–
	Tn-T (ng/L)	410.73 ± 22.41^*^	474.75 ± 23.66^**♯^	–	–
	BNP (ng/L)	126.42 ± 5.77^*^	147.83 ± 8.62^**♯^	–	–
	TNF-α (ng/L)	396.76 ± 16.05^*^	465.60 ± 21.55^*♯^	–	–
	IL-6 (ng/L)	92.49 ± 8.57^*^	121.59 ± 8.29^**♯^	–	–

Notes: The data are presented as the mean ± SD. (*n* = 5–6/group). *p < 0.05 and **p < 0.01, IRI or BNX versus sham; ♯*p* < 0.05, IMD^−/−^ mice versus IMD^+/+^ mice at the same time point after IRI or BNX.

Furthermore, histological examination of mouse kidney tissues *via* H&E and PAS staining (Figure 2E–G) revealed structural differences between the IMD^+/+^ and IMD^−/−^ sham groups. In the IMD^+/+^ sham group, kidney tissue exhibited a well-defined structure, characterized by densely packed tubular epithelial cells and neatly arranged brush borders. In contrast, the IMD^−/−^ sham group showed relatively sparse tubular epithelial cells, partial dilation of tubular lumens, and detachment of brush borders. Although the histological injury scores of the two groups did not reach statistical significance, these findings still suggest that even in the absence of overt kidney injury, IMD plays a role in maintaining kidney structural integrity. Compared with sham-operated controls, kidneys subjected to IRI exhibited significant pathological alterations at 24, 48, and 72 h post-IRI. These changes included varying degrees of kidney tubular epithelial cell swelling, vacuolar degeneration, loss of brush border, and focal necrotic detachment. Additionally, partial tubular luminal dilation with protein casts and cellular debris deposition was observed, accompanied by varying levels of inflammatory cell infiltration in the kidney interstitium. Among these time points, the 24 h group displayed the most severe kidney pathological damage. Notably, IMD^−/−^ mice exhibited a more pronounced increase in histopathological injury scores at each time point following IRI compared to their IMD^+/+^ counterparts. These experimental findings demonstrate that IMD plays a protective role in mitigating IRI-induced AKI.

### IMD Alleviates AHF following AKI

**Figure 3.**
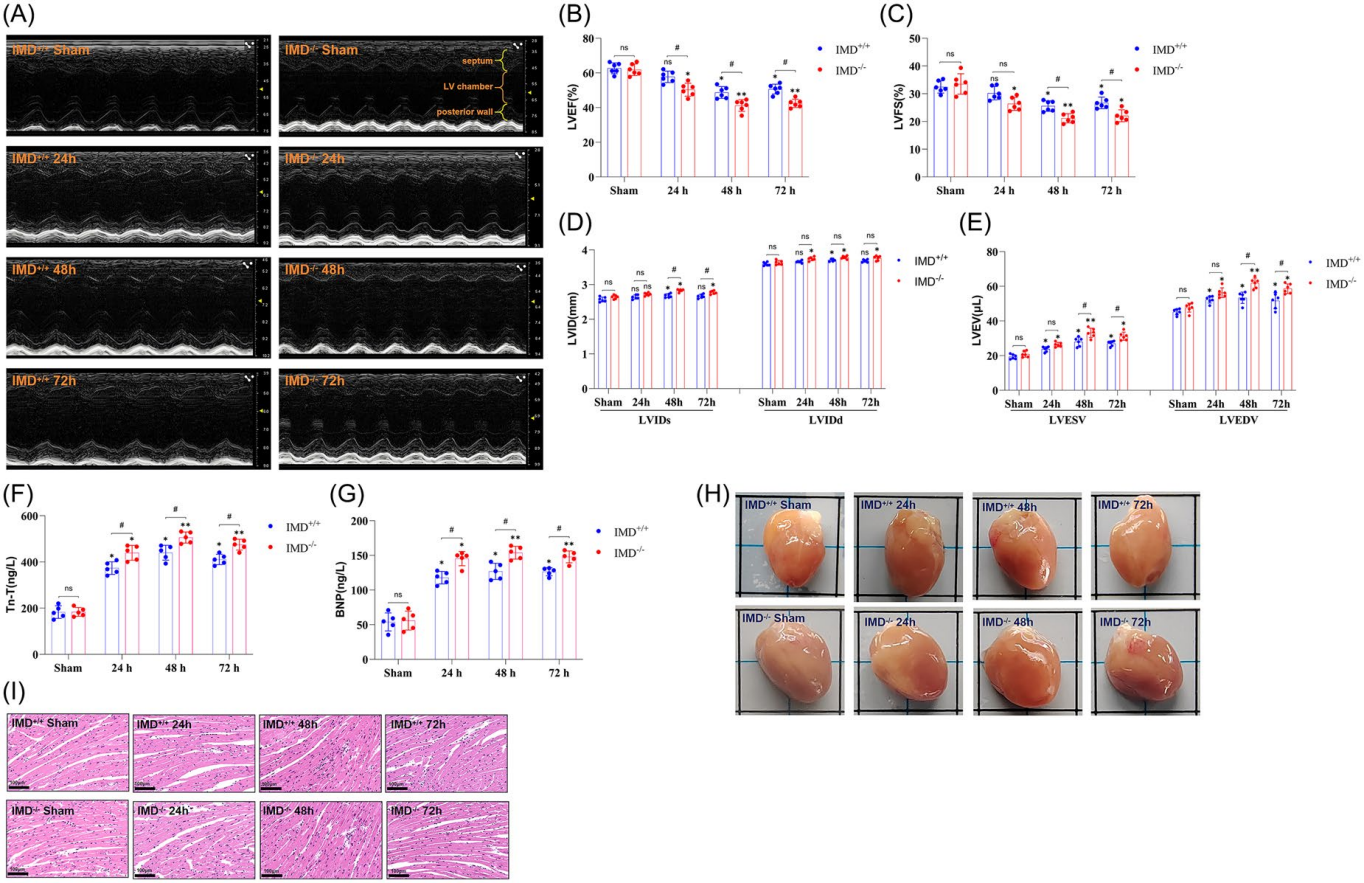
Cardiac injury in IMD^+/+^ and IMD^−/−^ mice after kidney ischemia-reperfusion injury. (A) M-mode echocardiograms of the left ventricle. (B) Left ventricular ejection fraction (LVEF). (C) Left ventricular fractional shortening (LVFS). (D) Left ventricular internal diameter at end-systole (LVIDs) and end-diastole (LVIDd). (E) Left ventricular end-systolic volume (LVESV) and end-diastolic volume (LVEDV). (F) Serum troponin T (Tn-T) levels in mice. (G) Serum brain natriuretic peptide (BNP) levels in mice. (H) Gross anatomical images of the heart. (I) H&E staining of the heart. The data are presented as the mean ± SD. (*n* = 5-6/group). ns: no significance; *p < 0.05 and **p < 0.01, IRI versus sham; ♯*p* < 0.05, IMD^−/−^ mice versus IMD^+/+^ mice at the same time point after IRI.

Compared with IMD^+/+^ sham mice, IMD^−/−^ sham mice exhibited no significant differences in echocardiographic indices. Notably, no direct surgical interventions were performed on the hearts throughout the experiment. However, echocardiographic assessments revealed alterations in certain cardiac parameters following kidney IRI (Figure 3A–E, Table 3, and Supplementary Table 4). Specifically, increase in LVIDs and LVIDd, as well as in LVESV and LVEDV were observed. Additionally, LVEF and LVFS were reduced compared to the corresponding sham group (*p* < 0.05). Among the time points examined, the 48-h group displayed the most pronounced echocardiographic changes. Moreover, IMD^−/−^ mice exhibited more significant alterations in cardiac parameters than IMD^+/+^ mice at the same time points (*p* < 0.05). These findings suggest that IMD mitigates certain echocardiographic abnormalities in mice following AKI.

**Table 3.** Statistical table of echocardiographic results for IMD^+/+^ and IMD^−/−^ mice.

	Sham		24 h		48 h		72 h	
Variables	IMD^+/+ ^mice	IMD^−/−^ mice	IMD^+/+^ mice	IMD^−/−^ mice	IMD^+/+^ mice	IMD^−/−^ mice	IMD^+/+^ mice	IMD^−/−^ mice
HR (BPM)	433.24 ± 27.92	426.32 ± 24.99	430.10 ± 34.38	435.40 ± 12.66	428.84 ± 22.63	418.20 ± 36.47	436.74 ± 30.37	442.83 ± 24.89
LVIDs (mm)	2.57 ± 0.29	2.64 ± 0.21	2.63 ± 0.16	2.72 ± 0.13	2.68 ± 0.09^*^	2.83 ± 0.07^*♯^	2.66 ± 0.17	2.77 ± 0.07^*♯^
LVIDd (mm)	3.59 ± 0.35	3.63 ± 0.16	3.66 ± 0.27	3.74 ± 0.06^*^	3.70 ± 0.06^*^	3.78 ± 0.10^*^	3.68 ± 0.15	3.76 ± 0.08^*^
LVESV (µL)	19.35 ± 1.03	20.84 ± 0.97	23.69 ± 1.51^*^	26.32 ± 1.31^*^	27.95 ± 1.69^*^	33.15 ± 1.21^**♯^	26.98 ± 1.50^*^	31.19 ± 1.72^*♯^
LVEDV (µL)	45.26 ± 4.67	47.70 ± 3.18	52.31 ± 6.87^*^	56.26 ± 3.45^*^	53.35 ± 4.58^*^	61.83 ± 2.92^**♯^	51.66 ± 2.70^*^	58.61 ± 2.74^*♯^
SV (µL)	27.58 ± 8.04	28.07 ± 4.26	28.55 ± 3.34	30.77 ± 4.54	29.50 ± 1.65	31.60 ± 2.53	28.72 ± 3.49	29.83 ± 3.37
LVEF (%)	62.69 ± 4.53	61.85 ± 4.64	57.74 ± 3.30	50.30 ± 3.74^*♯^	48.70 ± 3.37^*^	41.18 ± 3.35^**♯^	50.65 ± 4.53^*^	42.44 ± 4.06^**♯^
LVFS (%)	32.45 ± 3.11	31.49 ± 3.69	30.20 ± 2.41	26.35 ± 2.38^*^	25.55 ± 1.89^*^	21.15 ± 1.18^**♯^	26.69 ± 2.35^*^	22.03 ± 2.39^*♯^
CO (mL/min)	14.83 ± 1.32	13.56 ± 1.55	12.22 ± 0.83	11.00 ± 2.70	13.30 ± 1.89	12.89 ± 2.22	13.21 ± 1.13	11.46 ± 2.61
LV Mass (mg)	99.40 ± 16.64	101.41 ± 12.16	97.57 ± 15.56	104.65 ± 16.87	95.99 ± 18.97	103.16 ± 14.72	105.51 ± 10.94	101.74 ± 16.79
LV Mass Cor (mg)	78.91 ± 13.36	85.41 ± 9.57	81.68 ± 12.39	87.02 ± 15.22	78.57 ± 15.25	84.82 ± 13.74	91.29 ± 9.06	88.01 ± 17.18
LVAW; s (mm)	1.02 ± 0.30	1.07 ± 0.29	0.98 ± 0.17	1.05 ± 0.06	1.01 ± 0.04	1.04 ± 0.07	0.96 ± 0.16	1.05 ± 0.14
LVAW; d (mm)	0.78 ± 0.07	0.80 ± 0.16	0.76 ± 0.12	0.79 ± 0.10	0.76 ± 0.13	0.81 ± 0.10	0.75 ± 0.13	0.78 ± 0.17
LVPW; s (mm)	0.97 ± 0.09	1.05 ± 0.31	0.99 ± 0.04	1.02 ± 0.14	0.98 ± 0.06	1.00 ± 0.08	0.94 ± 0.10	1.01 ± 0.14
LVPW; d (mm)	0.68 ± 0.09	0.72 ± 0.29	0.69 ± 0.08	0.71 ± 0.10	0.65 ± 0.14	0.67 ± 0.08	0.70 ± 0.08	0.68 ± 0.11

Notes: The data are presented as the mean ± SD. (*n* = 6/group). *p < 0.05 and **p < 0.01, IRI versus sham; ♯*p* < 0.05, IMD^−/−^ mice versus IMD^+/+^ mice at the same time point after IRI.

No significant differences in Tn-T and BNP were observed between IMD^−/−^ sham mice and IMD^+/+^ sham group. Compared to their respective sham controls, both Tn-T and BNP levels were significantly elevated at 24 h, 48 h, and 72 h following IRI in both IMD^−/−^ and IMD^+/+^ mice (*p* < 0.05), with peak values observed at 48 h. Moreover, IMD^−/−^ mice exhibited significantly greater increases in Tn-T and BNP compared to IMD^+/+^ mice at the same time points (Figure 3F and G, Table 2, and Supplementary Table 4). These findings are consistent with the cardiac ultrasound results in mice, suggesting that IMD attenuates cardiac injury following AKI. These metrics were aggregated into forest plots in Supplementary Figs. 3 and 4.

Additionally, gross morphological examination revealed no significant differences in cardiac appearance between IMD^−/−^ and IMD^+/+^ mice under basal conditions or after kidney IRI (Figure 3H). However, histological examination of cardiac tissue *via* H&E staining (Figure 3I) demonstrated that sham-operated IMD^+/+^ mice exhibited well-organized myocardial cell structure with tightly connected fiber bundles and intact cellular alignment. In contrast, the sham-operated IMD^−/−^ group displayed partial blurring of myocardial cell striations, widened interstitium, and cord-like or reticular fibrous tissue hyperplasia within the interstitium. These findings suggest that even in the absence of overt cardiac injury, IMD plays a role in maintaining the normal pathological structure of the heart. Following kidney IRI, both IMD^+/+^ and IMD^−/−^ mice exhibited varying degrees of inflammatory cell infiltration in the myocardial interstitium and enhanced eosinophilic staining of cardiomyocytes (manifesting as a deeper pink coloration) at 48 h and 72 h compared to their respective sham-operated controls. Of particular significance, IMD^−/−^ mice exhibited more pronounced histological changes in cardiac tissue compared to IMD^+/+^ mice at the same time points following IRI. These findings underscore that kidney IRI can elicit inflammatory damage in remote cardiac tissue, and IMD appears to play a protective role in attenuating such injury.

### IMD Alleviates AHF following AKI through direct cardioprotection

**Figure 4.**
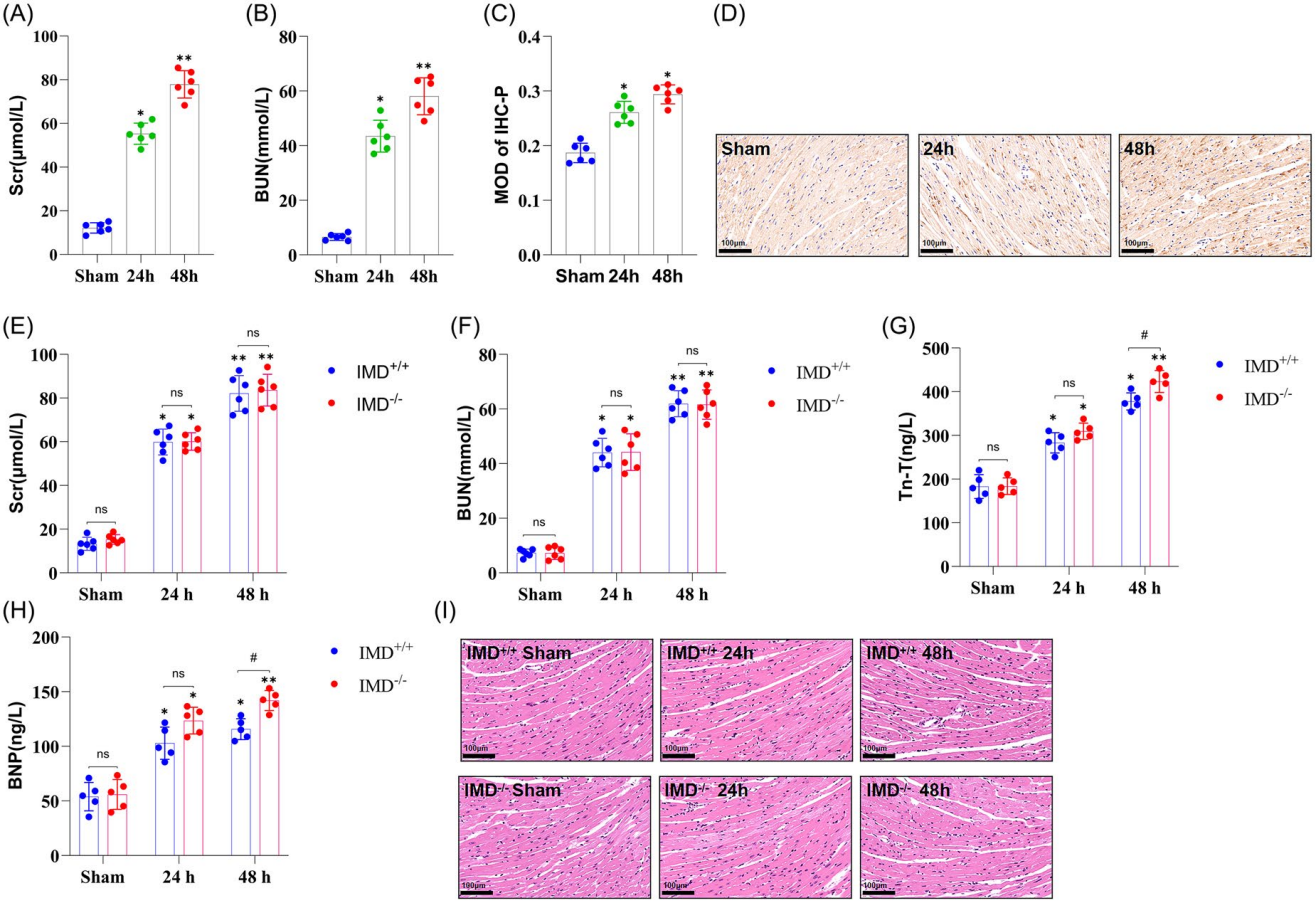
Cardiac injury in IMD^+/+^ and IMD^−/−^ mice after BNX. (A) Serum creatinine (Scr) levels in C57BL/6J mice; (B) Blood urea nitrogen (BUN) levels in C57BL/6J mice; (C) Semi-quantitative analysis of IMD immunohistochemical staining in the heart of C57BL/6J mice; (D) Representative images of IMD immunohistochemical staining in the heart of C57BL/6J mice; (E) Serum Scr levels in mice; (F) Serum BUN levels in mice; (G) Serum troponin T(Tn-T) levels in mice; (H) Serum brain natriuretic peptide (BNP) levels in mice; (I) H&E staining of the heart. The data are presented as the mean ± SD. (*n* = 5-6/group). ns: no significance; *p < 0.05 and **p < 0.01, BNX versus sham; ♯*p* < 0.05, IMD^−/−^ mice versus IMD^+/+^ mice at the same time point after BNX.

Because attenuating AKI itself lessens subsequent AHF, we sought to determine the direct cardiac effects of IMD independent of any nephroprotective action. To eliminate the potential confounding influence of AKI severity on cardiac outcomes, we established a BNX model as an alternative AKI model. Cardiac evaluations were performed at 24 and 48 h post-BNX. In C57BL/6J mice, Scr and BUN levels exhibited significant increases in the BNX groups compared to the corresponding sham-operated groups at both 24 and 48 h groups (*p* < 0.05; Figure 4A and B), with more pronounced elevations at 48 h than at 24 h. Consistent with our findings in the IRI-AKI model, immunohistochemical staining revealed increased IMD expression in cardiac tissues of BNX mice (Figure 4C and D), suggesting that IMD may be involved in cardiac injury and repair following BNX.

Kidney function did not differ significantly between IMD^−/−^ and IMD^+/+^ mice post-BNX (Figure 4E and F, Table 2). Serum Tn-T and BNP levels in both IMD^−/−^ and IMD^+/+^ mice (Figure 4G and H, Table 2) were markedly elevated in BNX groups compared to their respective sham controls (*p* < 0.05), confirming the successful establishment of AHF in these models. Higher Tn-T and BNP levels were observed at 48 h compared to 24 h, with IMD^−/−^ mice exhibiting greater increases than IMD^+/+^ mice at matched timepoints. Furthermore, H&E staining of cardiac tissues demonstrated more severe pathological damage in IMD^−/−^ mice compared to IMD^+/+^ mice post-BNX (Figure 4I). Collectively, these findings demonstrate that IMD alleviates AHF following AKI not only by mitigating the severity of AKI but also through direct protective effects on cardiac tissue.

Additionally, the postoperative survival rates of mice in the BNX model were analyzed (Supplementary Fig. 2). Compared with the sham group, both the IMD^−/−^ and IMD^+/+^ groups exhibited a significant decrease in survival rates at 24, 48, and 72 h postoperatively, with survival rates dropping to 0% at 72 h. Therefore, only mice at 24 and 48 h postoperatively were selected for further investigation. Moreover, changes in body weight pre- and postoperatively were monitored, revealing a significant decrease in body weight in both IMD^−/−^ and IMD^+/+^ mice following surgery (Supplementary Table 3). These findings indicate that BNX surgery has a substantial impact on the survival and body weight of mice, highlighting the necessity for meticulous surgical procedures and enhanced postoperative care.

### IMD Alleviates inflammatory response in cardiac tissue following kidney IRI

**Figure 5.**
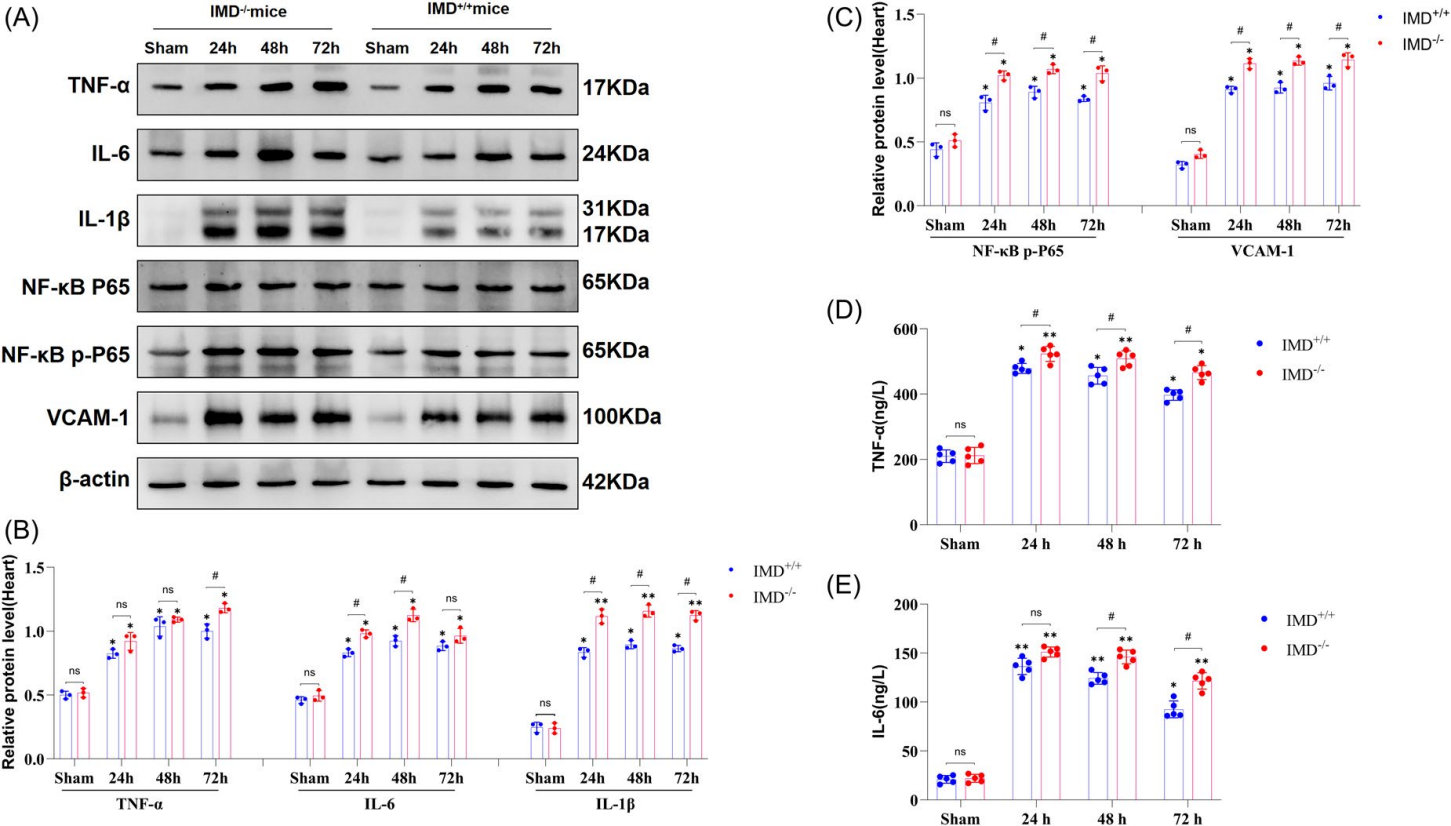
Heart and systemic inflammatory responses in IMD^+/+^ and IMD^−/−^ mice following AKI. (A) Representative western blot images of inflammation-related proteins in the heart; (B) Quantitative data analysis of TNF-α, IL-6, and IL-1β protein expression levels in the heart; (C) Quantitative data analysis of NF-κB P65 and VCAM-1 protein expression levels in the heart; (D) Serum TNF-α levels in mice; (E) Serum IL-6 levels in mice. The data are presented as the mean ± SD. (*n* = 5/group). ns: no significance; *p < 0.05 and **p < 0.01, IRI versus sham; ♯*p* < 0.05, IMD^−/−^ mice versus IMD^+/+^ mice at the same time point after IRI.

Given the pivotal role of inflammation in the progression of AKI-AHF, we investigated the mechanisms by which IMD mitigates post-AKI cardiac injury with a focus on inflammation. Given that IMD is an autocrine/paracrine small peptide, we investigated its impact on local cardiac inflammatory responses. Our results demonstrated that IMD significantly reduced the production of inflammatory factors in cardiac tissue of mice following kidney IRI (Figure 5A and B). Compared to IMD^+/+^ sham-operated mice, no significant differences in TNF-α, IL-6, or IL-1β protein levels were observed in the IMD^−/−^ sham-operated group. However, all three inflammatory factors were markedly upregulated at 24, 48, and 72 h in both IMD^+/+^ and IMD^−/−^ mice post-kidney IRI compared to their respective controls (*p* < 0.05). Among these time points, IL-6 and IL-1β exhibited the most pronounced upregulation at 48 h, while TNF-α showed peak elevation at 72 h. Notably, IMD^−/−^ mice displayed significantly greater upregulation of TNF-α, IL-6, and IL-1β compared to IMD^+/+^ mice at corresponding time points. These findings indicate that kidney IRI induces increased expression of inflammatory mediators in cardiac tissue, and IMD effectively attenuates this AKI-induced inflammatory response.

Furthermore, inflammatory factors can activate the NF-κB pathway, thereby amplifying the production of cytokine production, adhesion molecules, and chemokines and triggering inflammatory cascades. Our study revealed that IMD suppressed kidney IRI-induced activation of NF-κB and overexpression of VCAM-1 in cardiac tissue (Figure 5A and C). No significant differences in NF-κB p-P65 or VCAM-1 protein levels were observed between IMD^+/+^ and IMD^−/−^ sham groups. Both NF-κB p-P65 and VCAM-1 showed significant upregulation at 24, 48, and 72 h post-IR in both IMD^+/+^ and IMD^−/−^ mice compared to their controls (*p* < 0.05), with maximal NF-κB p-P65 activation at 48 h and peak VCAM-1 expression at 72 h. IMD deficiency resulted in significantly enhanced upregulation of these markers compared to wild-type counterparts at matched time points.

In summary, IMD exerts a protective effect against inflammatory damage in cardiac tissue following kidney IRI in mice. The underlying mechanism may involve direct suppression of inflammatory factors production within cardiac tissue or by inhibiting the activation of NF-κB in the heart, thereby reducing the production of cytokines and adhesion molecules.

### *IMD Alleviates systemic inflammatory response following AK*I

To further investigate the effects of IMD on systemic inflammatory response following AKI, we measured the serum levels of inflammatory cytokines TNF-α and IL-6 in mice subjected to different AKI models. No significant differences in TNF-α or IL-6 levels were observed between the IMD^+/+^ sham group and IMD^−/−^ sham group (Figure 5D and E, Table 2, and Supplementary Table 4). Compared to the sham group, TNF-α and IL-6 levels were significantly elevated at 24 h, 48 h, and 72 h post-kidney IRI (*p* < 0.05), with the most pronounced increase occurring at 24 h. Notably, IMD^−/−^ mice exhibited more significant increases in TNF-α and IL-6 compared to IMD^+/+^ mice at the same time points (*p* < 0.05). These findings collectively indicate that AKI induces systemic inflammatory responses, and IMD mitigates the elevation of circulating inflammatory cytokines following AKI.

## Discussion

In this study, we observed increased expression of IMD and its receptors in both kidneys and hearts following kidney IRI-induced AKI. Building on this observation, IMD^−/−^ mice exhibited more severe cardiac damage compared to their IMD^+/+^ littermate controls after kidney IRI, indicating that IMD alleviates AKI-associated AHF. In the BNX-induced AKI model, similar cardiac protection was observed where IMD^−/−^ mice developed more pronounced cardiac injury than IMD^+/+^ mice post-BNX, suggesting direct cardioprotective effects of IMD. Further studies have demonstrated that, following IRI-induced AKI, IMD^−/−^ mice exhibited a more significant upregulation of inflammation-associated proteins in cardiac tissue, as well as a more substantial elevation in serum inflammatory factor levels, compared with IMD^+/+^ mice. Collectively, our findings demonstrate that IMD mitigates AHF following AKI, potentially through attenuating both cardiac and systemic inflammatory responses.

### The impact of AKI on the heart

AKI, a systemic condition capable of inducing remote cardiac damage, significantly impacts clinical outcomes in affected patients [25,26]. Targeted interventions to mitigate cardiac injury following AKI may therefore constitute a crucial therapeutic approach for enhancing patient prognosis. In clinical practice, kidney IRI is a leading cause of AKI [27,28]. In basic research, the bilateral kidney IRI model has become the most prevalent animal model for investigating kidney-heart interactions. This model exhibits similar characteristics to the clinical impacts of post-AKI kidney injury and remote organ damage observed in clinical settings [12,29]. In this study, following kidney IRI, elevated levels of Scr and BUN were observed in mice, accompanied by significant pathological damage in the kidney. These findings indicate the successful establishment of the AKI model. Additionally, increased serum levels of BNP and Tn-T were detected following kidney IRI. Cardiac ultrasound revealed enlarged LVIDs and LVIDd, increased LVESV and LVEDV, and reduced LVEF and LVFS. These results suggest that AKI can induce remote cardiac injury.

### Post-AKI changes in cardiac IMD expression and its role in mitigating cardiac injury

IMD is a peptide that exerts cardioprotective effects. Studies have demonstrated its efficacy in alleviating heart failure induced by myocardial ischemia [30–32]. Similarly, in diabetic rats subjected to cardiac ischemic injury, treatment with IMD has been shown to ameliorate cardiac damage [33]. Furthermore, Zhang et al. reported that IMD could attenuate cardiac remodeling in mice [34]. However, the role of IMD in AHF induced by AKI remain unclear. In the present study, we initially assessed the expression levels of IMD and its receptors in the heart following kidney IRI. Our findings indicated that the mRNA and protein expression levels of IMD and its receptors were significantly upregulated in the heart post-IRI. Further investigation revealed that, following AKI, IMD^−/−^ mice exhibited more severe AHF compared to their IMD^+/+^ littermate controls. These results suggest that IMD may exert a protective effect against cardiac damage induced by IRI-AKI.

### Direct cardiac effect

In this study, we also observed that IMD alleviates AKI following kidney IRI, findings that are consistent with our previous work [35]. This observation raises the question of whether IMD exerts its protective effects on AHF indirectly by mitigating AKI or directly on cardiac tissue. To elucidate this, we employed a BNX-induced AKI model to eliminate the confounding effects of varying AKI severities on cardiac outcomes. Our results demonstrated that, following BNX, IMD^−/−^ mice exhibited more severe cardiac injury compared to their wild-type counterparts. These findings suggest that IMD can alleviate AHF following AKI through direct protective effects on cardiac tissue.

### Cardiac inflammation

Next, we investigated the potential mechanisms by which IMD ameliorates cardiac injury following AKI. Given that inflammation is a critical factor in the development of AHF after AKI [8,36], we focused on the inflammatory processes occurring in both the heart and the circulation. Previous studies have demonstrated that kidney IRI-induced AKI leads to the upregulation of pro-inflammatory mediators in cardiac tissue, thereby exacerbating the pathogenesis of AHF [12]. During AHF, the increased expression of inflammatory cytokines in the heart, such as TNF-α, IL-6, and IL-1β, is associated with higher rates of cardiovascular event rates and mortality [37–39]. Our data revealed that kidney IRI significantly upregulated the expression of inflammation-related proteins in the heart, including nuclear factor-κB p-P65, TNF-α, IL-6, IL-1β, and VCAM-1, in mice. These findings are consistent with prior reports [40,41]. Notably, pharmacological inhibition of cardiac inflammatory mediators has been shown to attenuate IRI-induced AHF [12].

IMD, a locally acting endocrine/paracrine peptide [20,21], has demonstrated robust anti-inflammatory properties across multiple disease models [42,43]. For instance, Li et al. reported that IMD significantly ameliorates cardiac inflammation in diabetic rats by inhibiting NF-κB activation and downstream cytokine expression [33]. This finding suggests a potential cardioprotective role for IMD in inflammation associated with AKI-related. To further investigate this, we utilized IMD^−/−^ mice and observed a marked exacerbation of cardiac upregulation of NF-κB p-P65, TNF-α, IL-6, IL-1β, and VCAM-1 following kidney IRI, compared to IMD^+/+^ controls. This indicates that IMD plays a crucial role in attenuating post-AKI cardiac inflammation. Mechanistically, our data support two non-mutually exclusive pathways: First, IMD may directly suppress the production of cardiac inflammatory mediators, which is consistent with its well-established tissue-protective effects [44]. Second, IMD likely inhibits the activation of the NF-κB pathway thereby reducing the synthesis of cytokines and chemokines and consequently dampening the inflammatory cascades [45,46]. This dual mechanism of action could synergistically mitigate cardiac damage induced by AKI.

### Systemic inflammation

Furthermore, experimental models of AKI have consistently demonstrated elevated levels of circulating TNF-α, IL-6, and IL-1, which exert direct detrimental effects on cardiac function [47]. Subsequent studies have shown that reducing serum levels of these inflammatory factors, such as TNF-α and IL-6, significantly alleviates cardiac injury following AKI [12]. In the present study, serum levels of TNF-α and IL-6 were found to increase more significantly in IMD^−/−^ mice compared to IMD^+/+^ mice following AKI. This suggests that IMD may mitigate AKI-induced AHF by suppressing the production and release of inflammatory mediators. However, the temporal changes in inflammatory mediators observed during the early phase of AKI-AHF in this study were not fully consistent with previous reports. This discrepancy may be attributable to variations in experimental conditions, such as core body temperature, anesthesia depth, and ischemia duration. Additionally, circulating inflammatory factors following AKI may originate not only from injured kidney and cardiac tissues but also from extra-kidney organs such as the liver and spleen [5,48]. Therefore, given its endocrine and paracrine functions, the mechanism by which IMD influences circulating levels of inflammatory factors in AKI-AHF requires further investigation to be fully elucidated.

### Mechanistic difference

IMD has also been shown to exert protective effects in various organ injuries, including myocardial ischemia [49]. In myocardial ischemia models, IMD primarily exerts its effects by enhancing coronary blood flow [50], reducing myocardial cell apoptosis [33], modulating myocardial energy metabolism, and improving microcirculation [51,52]. These effects are largely mediated through the activation of cyclic adenosine monophosphate (cAMP) and nitric oxide (NO) signaling pathways [53], which improve myocardial perfusion and reduce ischemic injury. In contrast, our study emphasizes the unique role of IMD in cardiac protection following AKI, with its cardioprotective effects primarily attributed to the alleviation of systemic and myocardial inflammation. This may be achieved through the inhibition of cardiac NF-κB activation, thereby reducing the production of cytokines and adhesion molecules. This suggests a significant mechanistic difference between IMD’s effects in myocardial ischemia and AKI-induced AHF. The specific protective mechanisms of IMD offer a unique approach to addressing the cardiac pathology induced by kidney injury.

### Notable discoveries

The mechanisms underlying the role of IMD in AKI have been preliminarily elucidated. From a translational perspective, IMD not only represents a promising therapeutic target for the management of AKI, but also holds potential as an effective intervention to prevent or attenuate the onset of AHF following AKI. Although no clinical studies to date have directly investigated IMD-based therapeutic interventions in patients with AKI or AHF, recent clinical data have shown that elevated plasma IMD levels are independently associated with an increased risk of major adverse cardiovascular events in patients with non-ST-segment elevation acute coronary syndrome [54]. This observation highlights the potential of IMD as a prognostic biomarker for cardiovascular diseases. Taken together, the mechanistic insights from our preclinical study, along with the prognostic associations observed in clinical settings, underscore the dual translational value of IMD as both a therapeutic candidate and a biomarker. Future research should focus on characterizing the pharmacological properties of IMD and evaluating the feasibility of therapeutic strategies, including IMD mimetics, receptor-selective agonists, gene therapy, or modulation of downstream targets, to bridge the gap between experimental findings and clinical application. Through multidisciplinary collaboration, our ultimate goal is to translate IMD-related discoveries into clinical practice, identify novel therapeutic targets, improve clinical outcomes in patients with AKI, and ultimately advance diagnostic and therapeutic strategies in critical care nephrology.

### Limitations and prospects

Our study provides a novel interpretative perspective on the impact of IMD on AHF following AKI. The findings indicate that IMD may alleviate AKI-induced AHF by mitigating inflammatory responses in both the heart and systemic circulation, thereby demonstrating its potential for comprehensive cardiac protection against post-AKI damage. From a clinical application perspective, IMD not only presents a novel therapeutic target for AKI management but also holds promise as an effective intervention for preventing and mitigating the development of AHF following AKI.

However, several limitations should be acknowledged in this study. First, cardiac-specific IMD-knockout models were not used in this study. Cardiomyocyte injury is a pivotal driver of AKI-induced acute heart failure. Owing to current technical and resource constraints, we were unable to use cardiac-specific IMD-knockout models (e.g. cardiomyocyte-restricted Cre-loxP) to isolate the direct cardiac effects of IMD. We therefore plan to generate these models in follow-up studies to definitively confirm IMD’s cardioprotective role in the AKI-AHF paradigm. Second, we did not employ mice with IMD overexpression or administer exogenous IMD to more fully elucidate the role of IMD in AHF following AKI. Future studies will address this by applying recombinant IMD peptide or lentiviral IMD overexpression in cardiomyocytes under AKI-mimicking conditions. Third, although our data implicate the NF-κB signaling pathway, we did not apply pharmacological NF-κB inhibitors or cytokine blockade *in vivo*. These complementary approaches will be incorporated into future *in vitro* studies to further substantiate the contribution of inflammatory signaling to IMD-mediated cardioprotection. Fourth, Beyond the NF-κB axis examined here, our earlier work has implicated cAMP/PKA and nitric-oxide signaling in IMD-mediated protection [35,55]. While these pathways were not directly interrogated in the present AKI-AHF model, their involvement remains plausible and will be systematically evaluated in forthcoming studies of AKI-induced cardiac dysfunction.

### Conclusion

Collectively, our data reveal that AKI triggers cardiac IMD up-regulation, which concurrently blunts systemic inflammation and exerts direct cytoprotection on cardiomyocytes, thereby sustaining cardiac function (Supplementary Fig. 5).

## Supplementary Material

Supplemental Material

Supplemental Material

Supplemental Material

Supplemental Material

Supplemental Material

Supplemental Material

Supplemental Material
