# Supplementary material for: Intermedin prevents acute heart failure following acute kidney injury by alleviating inflammatory responses
Source: Ren Fail. 2026 Jan 12;47(1):2610795. doi: 10.1080/0886022X.2025.2610795 (PMC12798669; doi:10.1080/0886022X.2025.2610795)
Supplement: Supplemental Material [file IRNF_A_2610795_SM2877.docx]

**Supplementary Table 1. Paller’ s Scoring Table**

| Scoring Items | Scores | Scoring Criteria |
| --- | --- | --- |
| Tubular Dilation | 0 | No dilation of renal tubules, normal morphology. |
|  | 1 | Mild dilation, with an increase in tubular diameter not exceeding 50% of normal. |
|  | 2 | Moderate dilation, with an increase in tubular diameter between 50% and 100% of normal. |
|  | 3 | Severe dilation, with an increase in tubular diameter exceeding 100% of normal. |
| Brush Border Loss | 0 | Complete and normal brush border on the surface of renal tubular epithelial cells. |
|  | 1 | Partial loss of brush border. |
|  | 2 | Most of the brush border is lost. |
|  | 3 | Complete loss of brush border. |
| Casting Formation | 0 | No casts in renal tubules. |
|  | 1 | Few casts, with fewer than 5 casts per high-power field. |
|  | 2 | Moderate number of casts, with 5-10 casts per high-power field. |
|  | 3 | Numerous casts, with more than 10 casts per high-power field. |
| Cell Flattening | 0 | Normal morphology of renal tubular epithelial cells; no change in cell height. |
|  | 1 | Mild flattening, with a reduction in cell height not exceeding 30% of normal. |
|  | 2 | Moderate flattening, with a reduction in cell height between 30% and 60% of normal. |
|  | 3 | Severe flattening, with a reduction in cell height exceeding 60% of normal. |
| Cell Shedding | 0 | No shedding of renal tubular epithelial cells from the basement membrane. |
|  | 1 | Few cells shed, with fewer than 5 shed cells per high-power field. |
|  | 2 | Moderate number of cells shed, with 5-10 shed cells per high-power field. |
|  | 3 | Numerous cells shed, with more than 10 shed cells per high-power field. |

**Supplementary Table 2. Overview of Antibody Indicators for Western Blotting**

| **Indicators** | **Dilution Ratio** | **Manufacturer** | **Catalog Number** |
| --- | --- | --- | --- |
| TNF-α Rabbit mAb | 1:1000 | Cell Signaling Technology | 11948T |
| IL-6 Rabbit mAb | 1:1000 | Cell Signaling Technology | 12912T |
| IL-1β Mouse mAb | 1:1000 | Cell Signaling Technology | 12242S |
| NF-κBP65 Rabbit mAb | 1:1000 | Cell Signaling Technology | 8242T |
| NF-κBP-P65 Rabbit mAb | 1:1000 | Cell Signaling Technology | 3033T |
| VCAM-1 Rabbit mAb | 1:1000 | Cell Signaling Technology | 32653S |
| Rabbit Anti-IMD | 1:1000 | Bioss | bs-2985R |
| Rabbit Anti-CALCRL | 1:1000 | Bioss | bs-1860R |
| Rabbit Anti-RAMP1 | 1:1000 | Bioss | bs-1567R |
| Rabbit Anti-RAMP2 | 1:1000 | Bioss | bs-11971R |
| Rabbit Anti-RAMP3 | 1:1000 | Bioss | bs-11972R |
| β-actin Rabbit mAb | 1:20000 | ABclonal | AC038 |
| anti-rabbit IgG (H+L) | 1:1000 | Beyotime | A0208 |
| anti-mouse IgG (H+L) | 1:1000 | Beyotime | A0216 |

**Supplementary Table 3. Statistical Table of Body Weight Changes in BNX-AKI Mouse Models**

| **Group** | | **Sham** | **24 h** | **48 h** |
| --- | --- | --- | --- | --- |
| IMD^+/+^ | Preoperative (g) | 22.75 ± 1.46 | 23.08 ± 1.36 | 23.12 ± 1.44 |
|  | Postoperative (g) | 22.00 ± 1.51 | 21.16 ± 1.41* | 19.83 ± 1.28** |
|  | Loss rate (%) | 3.30% | 8.32% | 14.23% |
| IMD^-/-^ | Preoperative (g) | 22.76 ± 1.54 | 22.98 ± 1.23 | 22.95 ± 1.12 |
|  | Postoperative (g) | 21.71 ± 1.49 | 20.81 ± 1.36* | 18.93 ± 1.27** |
|  | Loss rate (%) | 4.61% | 9.44% | 17.52% |

Notes: The data are presented as the mean ± SD. (n = 6/group). **p* < 0.05 and ***p* < 0.01, preoperative versus postoperative within the same IMD^-/-^ mice or IMD^+/+^ mice.

**Supplementary Table 4. Report exact p-values and confidence intervals for major comparisons (e.g., IMD^⁻/⁻^ vs. IMD^⁺/⁺^)**

| **Model** | **Indicators** | **Group** | **95% confidence interval (CI)** | | | | ***P***  **(IMD-/- vs. IMD+/+)** |
| --- | --- | --- | --- | --- | --- | --- | --- |
|  |  |  | **IMD+/+ mice** | | **IMD-/- mice** | |  |
|  |  |  | **Lower Bound** | **Upper Bound** | **Lower Bound** | **Upper Bound** |  |
| **IRI-**  **AKI** | **Scr (μmol/L)** | Sham | 10.105 | 16.434 | 13.023 | 17.579 | 0.210 |
|  |  | 24h | 43.978 | 52.497 | 53.220 | 60.999 | 0.026 |
|  |  | 48h | 32.689 | 42.771 | 40.072 | 55.005 | 0.035 |
|  |  | 72h | 26.745 | 33.113 | 33.790 | 43.980 | 0.021 |
|  | **BUN (mmol/L)** | Sham | 5.781 | 8.758 | 4.816 | 9.653 | 0.975 |
|  |  | 24h | 28.025 | 36.433 | 37.629 | 46.036 | 0.022 |
|  |  | 48h | 22.629 | 30.350 | 29.050 | 37.291 | 0.031 |
|  |  | 72h | 14.298 | 21.969 | 21.726 | 29.645 | 0.027 |
|  | **Tn-T (ng/L)** | Sham | 149.184 | 216.713 | 160.037 | 207.439 | 0.959 |
|  |  | 24h | 339.943 | 407.208 | 399.258 | 479.548 | 0.034 |
|  |  | 48h | 399.787 | 479.956 | 475.695 | 535.783 | 0.028 |
|  |  | 72h | 382.904 | 438.549 | 445.374 | 504.119 | 0.019 |
|  | **BNP (ng/L)** | Sham | 37.761 | 70.098 | 38.973 | 72.852 | 0.820 |
|  |  | 24h | 106.503 | 129.108 | 132.553 | 158.001 | 0.025 |
|  |  | 48h | 112.502 | 141.414 | 142.289 | 165.309 | 0.021 |
|  |  | 72h | 119.257 | 133.574 | 137.133 | 158.542 | 0.020 |
|  | **TNF-α (ng/L)** | Sham | 186.328 | 233.923 | 180.956 | 243.572 | 0.884 |
|  |  | 24h | 459.552 | 497.702 | 494.568 | 551.694 | 0.036 |
|  |  | 48h | 424.244 | 487.886 | 478.226 | 538.074 | 0.042 |
|  |  | 72h | 376.830 | 416.688 | 438.832 | 492.359 | 0.018 |
|  | **IL-6 (ng/L)** | Sham | 16.128 | 25.771 | 16.978 | 27.017 | 0.687 |
|  |  | 24h | 126.046 | 146.810 | 144.659 | 157.447 | 0.029 |
|  |  | 48h | 116.785 | 131.566 | 137.395 | 154.795 | 0.023 |
|  |  | 72h | 81.850 | 103.133 | 111.288 | 131.887 | 0.021 |
|  | **LVIDs**  **(mm)** | Sham | 2.499 | 2.642 | 2.587 | 2.701 | 0.094 |
|  |  | 24h | 2.578 | 2.699 | 2.677 | 2.766 | 0.071 |
|  |  | 48h | 2.623 | 2.740 | 2.793 | 2.869 | 0.022 |
|  |  | 72h | 2.608 | 2.723 | 2.727 | 2.822 | 0.027 |
|  | **LVIDd**  **(mm)** | Sham | 3.542 | 3.645 | 3.565 | 3.696 | 0.275 |
|  |  | 24h | 3.626 | 3.694 | 3.687 | 3.800 | 0.073 |
|  |  | 48h | 3.671 | 3.742 | 3.740 | 3.834 | 0.058 |
|  |  | 72h | 3.742 | 3.723 | 3.693 | 3.830 | 0.135 |
|  | **LVESV**  **(μL)** | Sham | 17.941 | 20.764 | 19.038 | 22.658 | 0.174 |
|  |  | 24h | 22.147 | 25.240 | 24.975 | 27.680 | 0.081 |
|  |  | 48h | 25.237 | 30.665 | 30.326 | 35.977 | 0.026 |
|  |  | 72h | 25.391 | 28.572 | 28.725 | 33.660 | 0.024 |

**continued from preceding page**

| **Model** | **Indicators** | **Group** | **95% confidence interval (CI)** | | | | ***P***  **(IMD-/- vs. IMD+/+)** |
| --- | --- | --- | --- | --- | --- | --- | --- |
|  |  |  | **IMD+/+ mice** | | **IMD-/- mice** | |  |
|  |  |  | **Lower Bound** | **Upper Bound** | **Lower Bound** | **Upper Bound** |  |
| **IRI-**  **AKI** | **LVEDV**  **(μL)** | Sham | 43.428 | 47.096 | 44.985 | 50.443 | 0.109 |
|  |  | 24h | 50.181 | 54.436 | 53.269 | 59.274 | 0.083 |
|  |  | 48h | 49.918 | 56.774 | 58.770 | 64.898 | 0.025 |
|  |  | 72h | 46.850 | 56.474 | 55.679 | 61.533 | 0.032 |
|  | **LVEF (%)** | Sham | 59.442 | 65.942 | 58.356 | 65.350 | 0.661 |
|  |  | 24h | 54.279 | 61.209 | 46.379 | 54.227 | 0.047 |
|  |  | 48h | 45.167 | 52.242 | 37.667 | 44.696 | 0.028 |
|  |  | 72h | 47.664 | 53.634 | 39.895 | 44.978 | 0.033 |
|  | **LVFS (%)** | Sham | 30.126 | 34.776 | 28.235 | 34.743 | 0.550 |
|  |  | 24h | 27.663 | 32.729 | 23.884 | 28.820 | 0.062 |
|  |  | 48h | 23.565 | 27.533 | 19.391 | 22.901 | 0.024 |
|  |  | 72h | 24.521 | 28.856 | 19.786 | 24.284 | 0.019 |
| **BNX-**  **AKI** | **Scr (μmol/L)** | Sham | 11.538 | 16.065 | 11.491 | 17.128 | 0.725 |
|  |  | 24h | 53.673 | 66.083 | 55.948 | 64.339 | 0.929 |
|  |  | 48h | 73.512 | 90.691 | 75.977 | 91.246 | 0.743 |
|  | **BUN (mmol/L)** | Sham | 5.868 | 9.215 | 6.646 | 9.435 | 0.569 |
|  |  | 24h | 38.605 | 49.506 | 37.184 | 51.239 | 0.965 |
|  |  | 48h | 56.916 | 66.914 | 55.999 | 67.209 | 0.917 |
|  | **Tn-T (ng/L)** | Sham | 152.997 | 210.326 | 162.781 | 205.742 | 0.845 |
|  |  | 24h | 254.368 | 311.592 | 285.888 | 332.572 | 0.084 |
|  |  | 48h | 353.566 | 402.090 | 392.058 | 454.526 | 0.031 |
|  | **BNP (ng/L)** | Sham | 40.822 | 66.674 | 39.902 | 68.861 | 0.930 |
|  |  | 24h | 84.589 | 121.211 | 108.287 | 138.701 | 0.074 |
|  |  | 48h | 103.858 | 127.578 | 130.592 | 153.236 | 0.023 |

**Supplementary Table 5. Table of Key Findings by Experimental Model and Timepoint**

|  | **IRI-AKI** | **BNX-AKI** |
| --- | --- | --- |
|  | **Sham, 24 h, 48 h, 72 h** | **Sham, 24 h, 48 h** |
| **Kidney** | Kidney injury was evident in the groups at 24 and 48 hours postoperatively, with the most pronounced damage observed in the 24-hour group. | Bilateral nephrectomy excluded the influence of kidney injury severity on cardiac damage. |
|  | ①In C57BL/6J mice, renal IMD gene expression was significantly increased postoperatively, with the most pronounced elevation observed in the 72-hour group. | ①Postoperatively, IMD^⁻/⁻^ and IMD^⁺/⁺^ mice exhibited consistent renal injury. |
|  | ②Scr and BUN levels were significantly elevated postoperatively, reaching 3 to 5 times higher than baseline values, with the most pronounced increase observed in the 24-hour group. | ②Postoperatively, Scr and BUN levels were significantly elevated, reaching 5 to 8 times higher than baseline values, with the most pronounced increase observed in the 48-hour group. |
|  | ③Histopathological examination of the kidneys via H&E and periodic acid-Schiff staining revealed evident pathological damage postoperatively. The observed changes included varying degrees of tubular epithelial cell swelling, vacuolar degeneration, loss of brush borders, and focal necrosis with cell sloughing. The most severe damage was observed in the 24-hour group. | ③The mortality rate in this model is high, with a 100% mortality rate observed at 72 hours postoperatively. |
| **Heart** | Heart injury was evident in the groups at 48 and 72 hours postoperatively, with the most severe manifestations observed in the 48-hour group. | Evident heart injury was observed at 48 hours postoperatively. |
|  | ①In C57BL/6J mice, heart IMD gene expression was significantly upregulated postoperatively, with the most pronounced increase observed in the 48-hour group. | ①In C57BL/6J mice, heart IMD gene expression was significantly upregulated postoperatively, with the most pronounced increase observed in the 48-hour group. |
|  | ②Postoperative echocardiographic indices were significantly altered: LVIDs and LVIDd were increased, LVESV and LVEDV were elevated, while LVEF and LVFS were decreased. The most pronounced changes were observed in the 48-hour group. | - |

**continued from preceding page**

|  | **IRI-AKI** | **BNX-AKI** |
| --- | --- | --- |
|  | **Sham, 24 h, 48 h, 72 h** | **Sham, 24 h, 48 h** |
| **Heart** | ③Postoperatively, levels of Tn-T and BNP were significantly elevated, reaching 2 to 3 times higher than baseline values, with the most pronounced increase observed in the 48-hour group. | ②Postoperatively, levels of Tn-T and BNP were significantly elevated, reaching 2 to 3 times higher than baseline values, with the most pronounced increase observed in the 48-hour group. |
|  | ④Histological analysis of the heart by H&E staining demonstrated evident inflammatory cell infiltration in the 48-hour and 72-hour groups postoperatively, with the most pronounced infiltration observed in the 48-hour group. | ③Histological examination of the heart by H&E staining at 48 hours postoperatively revealed mild inflammatory cell infiltration. |
| **Cytokines** | ①Postoperatively, the expression of cytokines in cardiac tissue was significantly increased. TNF-α showed the most pronounced increase at 72 hours postoperatively, IL-6 and IL-1β exhibited the most significant elevation at 48 hours postoperatively. | - |
|  | ②Postoperatively, the expression of inflammation-related proteins in the heart was significantly increased. NF-κB p-P65 exhibited the most pronounced increase at 48 hours postoperatively, while VCAM-1 showed the most significant elevation at 72 hours postoperatively. | - |
|  | ③Postoperatively, the circulating levels of inflammatory cytokines TNF-α and IL-6 were significantly elevated, with the most pronounced increase observed in the 24-hour group. | - |
