# Supplementary figures and images for: Intermedin prevents acute heart failure following acute kidney injury by alleviating inflammatory responses
Source: Ren Fail. 2026 Jan 12;47(1):2610795. doi: 10.1080/0886022X.2025.2610795 (PMC12798669; doi:10.1080/0886022X.2025.2610795)

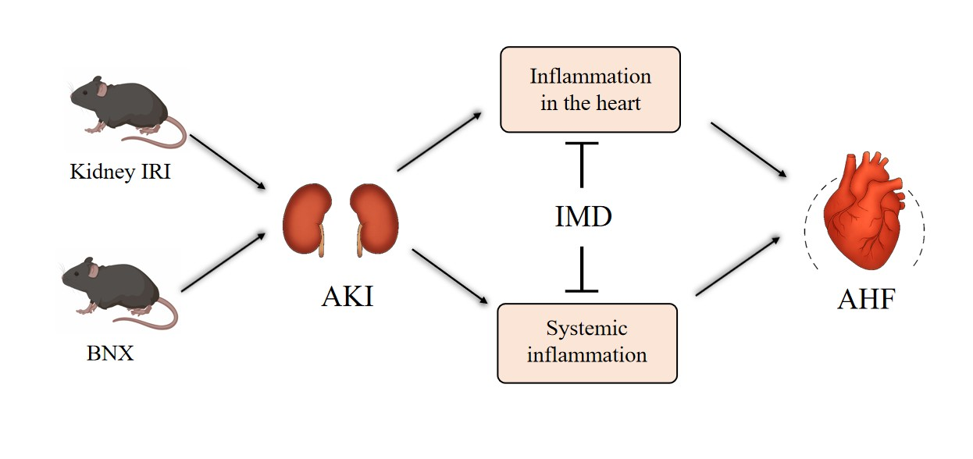

Supplement: Supplemental Material [file IRNF_A_2610795_SM2871.tif]

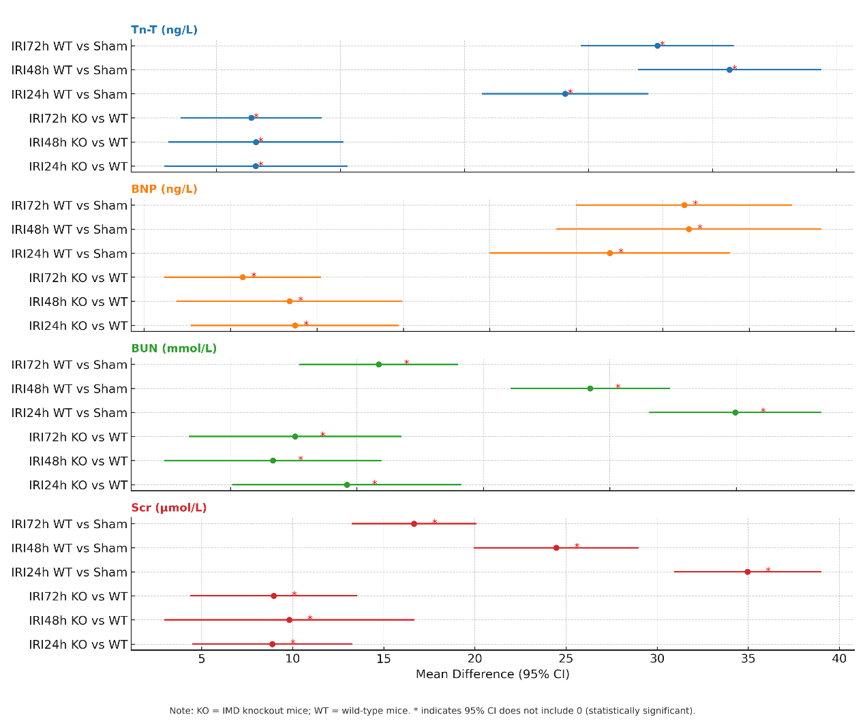

Supplement: Supplemental Material [file IRNF_A_2610795_SM2863.tif]

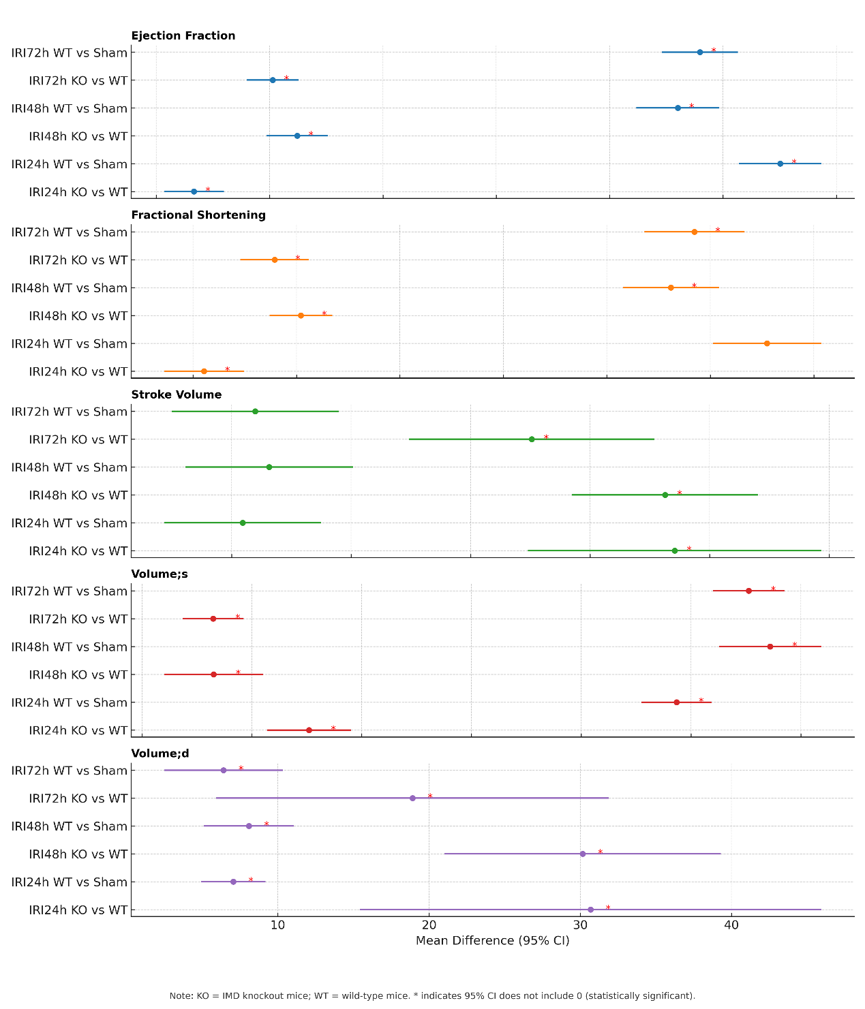

Supplement: Supplemental Material [file IRNF_A_2610795_SM2862.tif]

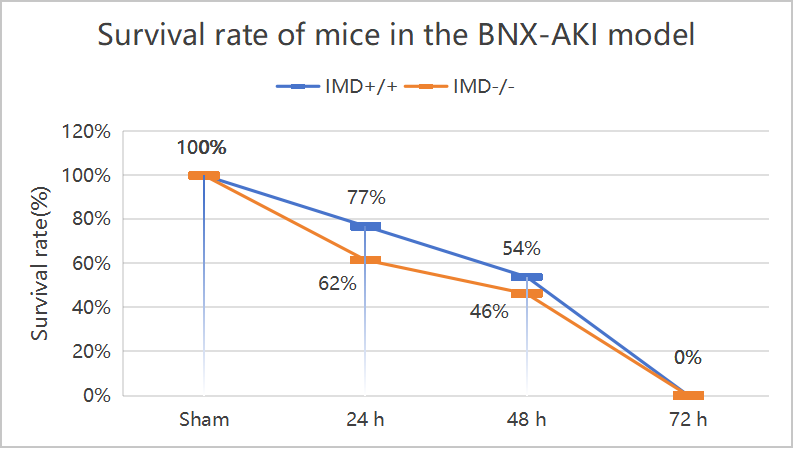

Supplement: Supplemental Material [file IRNF_A_2610795_SM2861.tif]

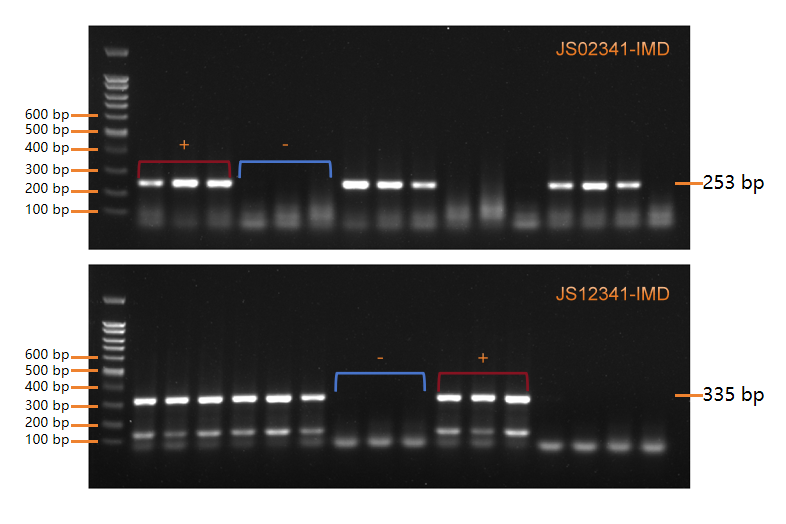

Supplement: Supplemental Material [file IRNF_A_2610795_SM2860.tif]
