# Supplementary material for: Intermedin prevents acute heart failure following acute kidney injury by alleviating inflammatory responses
Source: Ren Fail. 2026 Jan 12;47(1):2610795. doi: 10.1080/0886022X.2025.2610795 (PMC12798669; doi:10.1080/0886022X.2025.2610795)
Supplement: Supplemental Material [file IRNF_A_2610795_SM2870.docx]

**Supplementary Figure 1.** Gene identification results. The upper panel shows the electrophoresis results of JS02341-IMD; the lower panel shows the electrophoresis results of JS12341-IMD. The left panel displays the DNA Marker. Note: "+" indicates a positive result, "–" indicates a negative result.

**Supplementary Figure 2.** Survival rate of mice in the BNX-AKI model. The x-axis represents the time points, while the y-axis indicates the survival rate. The survival rate is calculated as the ratio of the number of surviving mice to the total number of mice. The total number of mice is equal to the sum of the surviving mice and the deceased mice.

**Supplementary Figure 3.** Forest plot-style figures of echocardiographic parameters in mice.

**Supplementary Figure 4.** Forest plot-style figures of serum biomarkers (Tn-T, BNP, Scr, and BUN) in mice.

**Supplementary Figure 5.** Schematic diagram of the dual protective mechanisms of IMD in AKI-AHF. Proposed causal mechanism linking AKI to cardiac protection via IMD up-regulation：AKI leads to the up-regulation of IMD expression in the heart, which contributes to both the suppression of systemic inflammation and direct cardiomyocyte protection. These actions ultimately help preserve cardiac function.
